# The Transactions of the Provincial Association

**Published:** 1836-10-01

**Authors:** 


### The Transactions of the Provincial Medical and Surgical Association. Volume IV. Octavo, pp. 578. 1836.

[Concluded from the last Number.]

The Provincial Society's volume arrived too late in the quarter to enable us to complete our notice of its contents in the last Number of this Journal. We gave a general sketch of their character, and pointed out those articles which were, and which were not adapted for analysis or criticism. Before we enter on our proper business, and consider separately each remaining paper, we would offer one or two brief remarks on the constitution of the volume.

It is too bulky; that is certain. None but the members of the Association would venture to purchase or peruse such a tome. Even the members of the Association itself must be actuated more by an esprit du corps, or by a positive engagement, than by their better judgment, if they deliberately expend their money or their time in wading through so many unprofitable pages. We will be bold enough to prophecy, that many a slumber will crown the tired country surgeon, as, dozing through whole prairies of topography, he sinks exhausted and bewildered in the midst of its monotonous details.

Addresses and anniversary proceedings possess but a local and a temporary interest. Animated by conviviality, perhaps by wine, the orator is received with favour and applause, and even were a critical examination possible, it would be out of place, injurious, and unkind. But such indulgence must die with the occasion, and the public, who did not partake of the dinner, care very little to hear the grace. At the festive board, the language of compliment is customary and pardonable. But when the harmless adulation is seriously offered to the attention of the world, an air of ridicule is necessarily thrown over both the proceedings and the actors.

Much of the present volume is not what a scientific society should publish, but matter only for a country newspaper. Ephemeral in its character, an ephemeral vehicle is best adapted for it, and as a speech is generally read with most avidity by the speaker or his more immediate friends, every reasonable object would be gained by its publication in the newspaper.

We would recommend the council of the Association to exercise a little gentle violence in compressing their succeeding volume. The addresses should be received in it with great precaution, or altogether rejected. The topographical surveys may be usefully abridged. Each article at present would appear to be as large as the place to which it relates, and the authors would seem to have imitated the stratagem of Dido, who cut out her hide with such ingenious minuteness, that she circumscribed within it the City of Carthage.* Perhaps the council would also do well to discourage papers of a speculative character. Such papers are usually indifferent, for although a man of brilliant talents may give them a high interest, if not a positive value, the generality of authors confer upon them neither. On the whole, we would direct the attention of the council to the plan pursued by that of the Medico-Chirurgical Society. Their Transactions offer an useful model to their provincial brethren.

While we feel disposed to object to part of what the Provincial Association have inserted, we must also express regret for what they have omitted. The exact taste of the day has created a demand for authentic facts. The most authentic in medicine are those of a clinical character—the cases which occur publicly in the hospital or the infirmary. The provincial hospitals are numerous, well organized, and well officered. The Transactions should be made the vehicle of diffusing a knowledge of the valuable facts that must

---

* There is an article of upwards of a hundred pages on the topography of the Land's-End. It is the second on the same subject, and we are promised a third. It is fortunate that it is the *Land's-end* at which the author has arrived.

constantly be noticed in them. Well arranged and systematic hospital reports should form a staple article in a volume like the present, and would materially enhance its utility and value. There are indeed some clinical reports in this, but they are not by any means sufficiently numerous.

Such are the observations we would offer. They are made with the view of raising the character and enlarging the utility of the Association's Transactions. We are anxious to forward the real interests of medical science, and perhaps there are no more gratifying, nor indeed effectual means, than by elevating the attainments, and consequently the station of provincial medical men. The increase of education, and Associations, such as are formed and forming, will, we feel convinced, be of essential benefit to the profession; education teaching them to think—Associations enabling them to act.

The first paper which now comes before us is one from Mr. Chavasse, a surgeon of Birmingham, on—

### Some of the injurious Effects of the Secale Cornutum.

The object of Mr. Chavasse is to shew, that the secale cornutum, although an useful and important medicine, is calculated, when injudiciously exhibited, to destroy the vitality of the fœtus, or to occasion hour-glass contraction of the uterus, and retention of the placenta. His observations are founded on the experience of practitioners of his acquaintance, as well as on his own, and apply more particularly to first labours. Although he has seen, or has been made acquainted with the particulars of several cases, he relates those of only two.

*Case* 1. Mrs. R. æt. 29, a delicate woman of nervous irritable temperament, was taken in labour at the full period of utero-gestation. Nothing occurred worthy of observation till she had been in labour between eight and nine hours, when the pains became very frequent, but inefficient. The os uteri was dilated to the extent of a five-shilling-piece. With a view to control this irritable state of the uterus, a warm emollient injection was administered (though the bowels had been previously unloaded), and a full dose of opium was given. These measures were productive of no benefit, and, in an hour afterwards, Mr. C. found her much exhausted, and the labour little advanced. Mild stimulants were had recourse to, but the patient became very faint after each pain. At this period the child was lively, the os uteri still about the size of a crown-piece, its edges thinner, the lochial discharge free, and the external parts disposed to yield. Thinking that the ergot would overcome the irregular and inefficient action of the uterus, Mr. C. gave the patient two scruples of the recently powdered substance in a wine-glass full of water. In the course of about twenty minutes, she began to complain of great pain and uneasiness in the hypogastric region. The pains increased in frequency and force, until they became continuous and violent, lasting fully half an hour, when they ceased altogether, and the patient grew restless and anxious.

"From this period the motions of the child were no longer perceptible; and on examination I found the labour but little advanced. Mrs. R. being now free from pain and much exhausted, fell asleep, and full two hours elapsed before there was any return of uterine action. An increased flow of lochial discharge was soon followed by some sharp bearing-down pains, and soon afterwards by the expulsion of a fine still-born child. Firm and steady pressure was made over the region of the uterus, but no attempt was made on the part of the womb to dislodge the placenta. As there was no hæmorrhage I waited nearly an hour, but still the uterus exhibited no disposition to throw off its contents, and my patient's, as well as my own anxiety being well awake, I introduced my hand, and immediately detected well-marked hour-glass contraction; the uterus being divided into two chambers, and the placenta lodged in the upper. Finding the stricture very firm, I withdrew my hand, and giving her a full dose of laudanum, waited ten minutes, and again introduced it. By steady pressure, with the fingers reduced to a conical shape, I soon overcame the annular stricture, and brought the placenta away in my hand. The uterus immediately contracted firmly, and nothing occurred to interrupt the convalescence of my patient." 309.

*Case* 2. At 5, a. m. of the 5th of June, 1830, Mrs. H. æt. 36, a delicate woman, was taken, at the full period, with slight uterine pains, and other indications of approaching labour.

"When I saw her," says our author, "at twelve o'clock, she was progressing slowly but satisfactorily, and the os uteri was opened to about the circumference of a shilling. I was called to her at six p. m., the pains being sharper, and the os uteri the size of a half-crown. The presentation was natural. The pains were slow, but regular, till twelve o'clock, when, increasing in frequency till two o'clock, the liquor amnii escaped, and there was a suspension of pain for three-quarters of an hour: the child being lively and troublesome. The circumference of the os uteri was now equal to that of a crown-piece, and its edges, though firm, yielded to pressure. I administered two scruples of powdered ergot. In twenty minutes it began to manifest its power, by producing pain in the lower part of the abdomen and back, which soon became severe, and continued without intermission for full half an hour, when she became quite easy. During this time there was but little bearing-down sensation, and I found, on examination, that neither the os uteri nor perineum had undergone any material change. She continued free from pain for nearly an hour, enjoying, in the interval, a little refreshing sleep. She was awaked by a return of the pains, which soon becoming severe and sensibly effective, the perineum was speedily on the stretch, and in less than half an hour my patient was delivered of a fine still-born child. After waiting full half an hour, the uterus in the interim shewing no disposition to throw off its contents, (although the belly and loins had been well rubbed, and the womb itself forcibly grasped from time to time with the hand, but without the effect of producing satisfactory contraction of the organ) my patient began to flood considerably. Unable to trace the insertion of the funis into the placental mass, I suspected there was hour-glass contraction, and on introducing my hand my suspicions were confirmed. I immediately withdrew it, gave her fifty drops of tincture of opium, and after waiting half an hour again passed my hand into the cavity of the uterus. The constriction was soon overcome; and as there was no adhesion, the after-birth was easily brought away. My patient ultimately did well, but no conception has since taken place." 311.

Mr. Chavasse makes several observations, and cites the expressed opinions of three of his medical friends, in order to shew that such consequences as have been described in the preceding cases, not uncommonly follow the exhibition of the ergot. It is unnecessary for us to dwell upon the subject, for the facts speak of themselves. There can be little question that so powerful an agent should be given with much caution, and that, in first labours more particularly, the surgeon should pause before he stimulates the uterus to forcible contraction. The relation of these facts will tend to put other practitioners upon their guard.

Mr. Chavasse cites the recommendations and conclusions of Dr. Dewees, with reference to the cases requiring the exhibition of the ergot. We may extract them.

" 1. It should never be given before the membranes are ruptured, the os uteri dilated, and the external parts disposed to yield.

2. But should the pains flag from any cause, it may be given, provided the labour be natural, according to the acceptation of the term natural labour—that is, when the head is well situated, the breech, the feet, or the knees present. For independently of any accident which may complicate the labour, it is desirable to hasten it sometimes, for the safety of the child, when the natural powers are incompetent to this end.

3. And if the labour be accompanied by any such accident as flooding, convulsion, syncope, &c. it may be sometimes employed to great advantage, provided rules 1 and 2 are not violated.

4. It may be used often with much advantage in every kind of premature labour; and at the full time, when the placenta is not thrown off, and the uterus is in a state of atony.

5. When floodings take place after the rupture of the membranes, the os uteri well dilated, the pains feeble, but the child well situated.

6. When the head of the child has been left in the uterus by being separated from its body.

7. When the uterus is painfully distended by coagula." 318.

Mr. Chavasse, however, is inclined to differ from Dr. Dewees, in the 4th rule that he lays down. His own observation leads him to conclude that the ergot exercises no power over the uterus, until the full term of pregnancy is accomplished, or till the muscular fibres of the organ have been excited to action by the pains of labour. He has tried it as an emmenagogue, in apparently appropriate cases, without benefit, and in two instances where it was given, in order to induce premature labour, it failed. But we may observe that some discrepancy of opinion exists on the power of the ergot in these cases, and there seems some ground for believing that it is far from absolutely inefficacious in producing premature contraction of the uterus.

Mr. Chavasse appends some observations on the efficacy of the ergot in uterine hæmorrhage occurring during labour. We will notice them as briefly as possible.

Mrs. Adkins, æt. 38, of a full plethoric habit, and the mother of seven children, was seized, at the full period of pregnancy, with slight labour-pains, followed by profuse hæmorrhage. When Mr. C. arrived, she was much exhausted; the pains had nearly disappeared; the os uteri was larger than a half-crown-piece, and dilatable, and on one side a portion of protruding placenta could be distinguished. Mr. C. ruptured the membranes, and, as the presentation was natural, gave her two scruples of the ergot in a wine-glassful of brandy and water. No more bleeding occurred, and in thirty minutes she was safely delivered of a child so putrid that it had evidently been dead for some days before the labour commenced.

The next case was that of a delicate woman, the mother of six children.

After being busily engaged till one o'clock in the morning, she retired to bed, and at two o'clock was awoke by symptoms of slight labour, and a most profuse hæmorrhage. When Mr. C. saw her, he found a considerable portion of the placenta protruding between the fœtal head and the os uteri, which was dilated to the size of a five-shilling-piece. The pulse was extremely feeble, the extremities were cold, and the patient altogether much exhausted. Mr. C. gave a drachm of the powder in some brandy and water, and the membranes, which were unusually thick, were then ruptured. In the course of half an hour, there was neither pain nor hæmorrhage. Hot brandy and water were then given, the pains returned, a still-born child came away, and in fifteen minutes the placenta followed.

Passing over another case, we will conclude with one intended to exhibit the good effects of cold water injected into the uterus.

*Case.* Mrs. B. a thin phlegmatic woman, æt. 28, was delivered of her first child after a natural labour of six hours' duration. The placenta came away, without assistance, in twenty minutes afterwards, and a tremendous hæmorrhage succeeded. Mr. C. desired the nurse to make pressure on the hypogastrium, while he introduced his hand into the cavity of the womb, to induce it to contract. It was loose and flaccid, and the attempt was ineffectual. The patient was deprived of animation. The ergot and brandy were administered in vain. The apparatus for the performance of transfusion was sent for, and was on the point of being used, when, having a half-pint syringe at hand, Mr. C. injected eight ounces of cold water into the cavity of the uterus. The effect was instantaneous. The organ instantly and powerfully contracted, reaction succeeded, and the patient was saved.

### II. Extensive Disease of the Female Pudendum. By *T. Brayne*, Esq. Surgeon, Banbury.

Mrs. S. æt. 39, when about 27 years of age, first observed a pendulous substance, of the size of a small nut, growing near the upper part of the left labium pudendi. In two or three years it had attained the size of a goose's egg, when it became more painful and grew faster. In the course of five or six years, it had attained nearly its maximum of size, and was accompanied by frequent, severe, and lancinating pains. In ten years from its commencement, it became deeply ulcerated in two places, and from one of them it twice bled profusely. The second time was a few months prior to the present report, and Mr. Brayne was then summoned as the patient was thought to be dying. Nothing had been done for the complaint, with the exception of the patient having herself applied a ligature, when it was small. Mr. Brayne's description of the present state of the tumor is as follows :—

"On examination, the tumour is found to grow by a neck, which increases to about five inches in circumference, and seems to be composed of both nymphæ, continuous at the upper part, and doubled together, for on raising the tumour against the abdomen and separating this duplicature, in its concavity is seen the orifice of the urethra in its natural situation, but the clitoris is undistinguishable. The tumour is flaccid and rather soft, and somewhat of a pyriform shape, with the largest end upwards. It measures fourteen inches and a quarter in circumference at its widest part, and ten a half in length, exclusive of the neck by which it grows, the lowest part of it reaching down, when hanging and unsupported, nearly to the knees. The general aspect of its surface is rough and tuberculated, in some parts being fissured and rugous, in others composed of prominent and clustered lobules of various sizes and shapes. The rugous, and apparently the oldest, portions are of a very dark brown colour, speckled by patches of cuticle of an intense black. The smaller tubercles are of a pale brown, while the larger and more distinctly lobulated portions are of a light red and more vascular appearance, and some are almost semi-transparent. Here and there are deep excavations, where ulcerations have existed and healed, one of which is seen, smooth and deep, near the centre of the mass. The ulcers that exist at present are smaller, and situated upon the posterior part of the tumour. Some of these have a yellow colour, like unhealthy granulations, from which there is constantly discharged an immense quantity of an offensive, irritating ichor, which is often tinged with blood. The whole skin of this mass possesses a considerable degree of sensitiveness, and large venous trunks ramify on its neck, and are soon lost in the body of the tumour. Ulceration of these vessels probably furnished the profuse discharge of blood that I witnessed, which was evidently venous, and amounted at least to four pints. On weighing the tumour, with as much care as possible, to ensure accuracy, it was found to weigh (Feb. 18th, 1835) full eleven pounds.

The posterior parts of the labia, the perineum, and the skin surrounding the anus for several inches, are also in the same lobulated and tuberculous state. One small, hard, flat tumour projects beyond the rest, about an inch and a half in length, from the margin of the anus; another, of the size of a walnut, occupies the orificium vaginæ, and grows by a neck from the posterior surface of that canal. All these parts partake of the same general character of disease as the large tumour; but they are, for the most part, more solid and resisting, like ordinary scirrhus. Much lancinating pain is constantly experienced in them; and there is, also, frequently discharged from the anus, a considerable quantity of the same offensive matter as from the ulcerated portions of the larger tumour." 372.

Her constitution appears to be giving way. She is without appetite, has a quick pulse, and impeded respiration.

We extract this case, because we think that it enables us to make an observation that may be useful on another occasion. It is quite clear that nothing can be done by an operation here. The extension of the disease to the vagina, perinæum, anus, and neighbouring integument, puts the employment of the knife out of the question.

But, if we understand Mr. Brayne's description rightly, we do not agree with him in his opinion. He conceives that the disease is essentially malignant, and a species of carcinoma. We have seen several instances of, apparently, a similar morbid growth from the female pudendum, in an earlier stage than, unfortunately, this is now. The tumor had the same slow growth, the same lobulated appearance, the same disposition to ulcerated fissures, the same abundance of large veins ramifying in it. But then, we say, it was in an earlier stage and of lesser size. The tumor was removed by the knife with success. It did not re-appear in the part, nor did other organs become implicated. On dissection of the tumor, it was found to be a production, apparently hypertrophic, of the skin and the subcutaneous adipose and cellular tissues. We repeat, that we have seen the operation performed with success on several occasions, and in the latter part of last year, we removed from the anterior commissure of the labia and the mons, a growth of this description ourselves.

We do not, therefore, believe that the tumor is malignant, in the sense of either originating in a constitutional taint, or, in general, of itself contaminating the system. If it is removed at a tolerably early period, that is, while its connexions and situation render it removeable, the surgeon need not be apprehensive respecting the result. A very wide difference this from the results of the operations for scirrhus and medullary sarcoma in their very earliest stages. But, when this tumor has lasted so long and spread so widely as in Mr. Brayne's patient, then it is not only locally beyond the reach of the knife, but probably it has assumed the malignant character, and other organs have become affected.

### III. Case of Neuralgia of the Uterus during Pregnancy. By *J. Holbrook*, M.D. Crickowell.

This is, in many respects, an interesting case. But it is long and the details are confused. The author does not appear to be much versed in that analytical method, which arranges and condenses the materials submitted to it. We shall endeavour to make it rather less involved than it is in its original form.

*Case.* On the 14th of March, 1835, Dr. Holbrook was summoned to Mrs. S. He found her labouring under symptoms closely resembling those of labour. The pains recurred every ten or fifteen minutes, and continued about ten minutes. They were felt in front, in the back, and extended down the hips. The pulse was quick, the skin was warm. These symptoms had appeared suddenly a few hours previously, accompanied by a considerable red "show." The patient had also been sick.

The history of the case was this. The lady had been, from a child, extremely delicate, and, since the age of puberty, had suffered from what were deemed frequent inflammatory attacks,* principally of the chest, for which she had been largely and repeatedly bled. Before her marriage, and afterwards, she had evinced a morbid sensibility in both the external and internal organs of generation; and had laboured under retention of urine, requiring the employment of the catheter, which, however, occasioned excessive pain. The medical practitioner who attended her at this time, makes the following statement:—

"Some time in 1830, before her marriage, Mrs. ——, after several inflammatory attacks, for which she had been largely and repeatedly bled, and when she was still confined to her bed, began to complain of great pain in the pelvic region, accompanied with leucorrhœa, particularly when the bowels were moved. This state was soon followed by spasms of the abdominal muscles, and severe

* It is probable that these "inflammatory attacks" were more of a nervous character than was supposed. It will appear in the sequel, that the patient laboured under those symptoms, which, for want of a better name, we call hysterical—symptoms which ape local inflammations with most deceptive closeness. But this is par parenthèse.

bearing down like the last stage of labour, occurring regularly once every twenty-four hours, and resisting every remedial measure, until strong opiate suppositories were had recourse to. The uterus was suspected to be in fault, but it was long before she would consent to an examination; and when that took place, the cervix of the organ was found to be in a high state of sensibility, the most gentle touch on examination producing excruciating pain, followed by syncope." 330.

When she married we are not informed, for the whole case is told in a rambling manner, the author speaking a good deal in lutestring. It appears, however, that in a short time after marriage she began to be affected with repeated attacks, threatening abortion, similar to that for which our author was summoned, and relieved by bleedings and opiate suppositories. And here we may observe that the bowels were habitually costive, and that opium could not be borne on the stomach in any shape, being always followed by severe vomiting, and general distress.

Thus she went on till the ninth month of her first pregnancy, in the first week of which she was brought to bed of a dead child, this being supposed to have perished about the end of the sixth month. This was in May. After it, she recovered her usual health, again became pregnant, and miscarried about the Christmas preceding the March in which our author first saw her.

Such is the connected history of the case, and, having glanced at it, let the reader revert to the state in which he and we found the patient on the 14th of March, when she was supposed to be about six or seven weeks advanced in pregnancy again.

After weighing all the circumstances, Dr. Holbrook was disposed to do nothing; but having waited half an hour, and not finding any progress made towards abortion, he abstracted eighteen ouncess of blood from the arm, and exhibited a suppository of opium. The bleeding was immediately followed by faintness, and a state of reverie, which continued for a considerable time; after which the pains gradually became less urgent, and the intervals longer; yet they lingered about the back and uterine region for at least twenty-four hours, before the process of utero-gestation seemed to be free from interruption.

This attack was now subdued, and our author did not again see the patient till the succeeding May, towards the end of which month another attack of the same kind took place, which was relieved by similar treatment; as were also the succession of attacks which took place at intervals of about three weeks, until the sixth of August, when she was prematurely confined of a dead fœtus. The recumbent posture, strictly maintained, light tonics, and gentle aperients, were the principal means adopted in the interim. In June she had an attack of retention of urine, and the introduction of the catheter produced such suffering, that each time it was succeeded by "a delirious reverie" that lasted several hours.

After the expulsion of the dead child in August, she had again, for some days, retention of urine, but otherwise she seemed to be doing pretty well. On the 24th, however, eighteen days after her confinement, a febrile state ushered in phlegmasia dolens, first on one side, then upon the other. The veins were distended, and uneasiness was experienced in their course, the swelling was far from very great, and the pulse was irregular and intermittent. There was no other symptom of affection of the heart.

Such are the facts of this interesting because not very uncommon case. When we use the terms not very uncommon, we do not mean that *all* the features are common—far from it; we merely intend to observe that the general characters of the case are not unfrequent. It is an instance of the multiform ailments, that arise from irritation or irritability of the uterine system. Sometimes *that* produces, as in this instance, symptoms directly referable to the generative organs—often it occasions sufferings in parts remote and not obviously connected with them. One female of this description will have excited sensibility of the uterus itself—"the irritable uterus;" a second will have simple hysteria; a third catalepsy; a fourth, morbid states of sensation in various regions of the body—pain in the chest, or the hypochondrium, or the breast, or the joints; a fifth, affection of the muscles of voluntary or involuntary motion—for example, chorea, wry neck, retention of urine, various forms of paralysis; a sixth, will exhibit a morbid condition of the mind, extraordinary tastes, altered affections, or a perverted morale.

All these are the offspring of that nervous condition of system, essentially connected with disturbance of the generative organs, and commonly called and considered hysterical. As its symptoms, or effects, are varied, so are the conditions on which it depends. Probably in the first instance, the functions only of the parts are deranged, and excitement of the ovaria or the uterus constitutes the efficient cause. But that excitement, attended as it is with periodical or permanent vascular action, lays the foundation of positive physical changes. Those changes are numerous, though seemingly minute. The most frequent is, no doubt, chronic inflammation and thickening of the cervix uteri. Sometimes fibrous tumors are developed in the substance, or on the surface of the uterus—sometimes polypi originate in its interior—sometimes cysts or other morbid productions occur in the ovarium; in short, the physical changes that ensue from morbid excitement of the uterus and the ovaria are very various and very vague.

It is too frequently the practice to set down to the account of mere fancy and caprice, the extraordinary symptoms of nervous females. Occasionally they arise from an absence of due control over the feelings or the passions. But frequently positive physical changes in the generative organs produce them, and it is equally unjust and cruel to suppose that mere efforts of the mind can put them down. The practical physician is well aware that positive affections of the organs of digestion are constantly the source of hypochondriasis, and that alterations of the brain are the cause of monomania. Yet it is not very long since the mind, an abstract metaphysical entity, was said to be exclusively in fault, and to morbid anatomy we owe the correction of this dangerous error. The case of hysteria is analogous to that of monomania and of hypochondriasis. Physical conditions of the uterine system, sometimes positive physical changes, give birth to its heterogeneous phenomena. And the object of the intelligent practitioner should be to determine in the individual case, whether any changes have occurred or not.

We have been led to make the preceding observations from having, on more than one occasion, seen actual disease of the uterus and its appendages overlooked, and symptoms dependent on it attributed to mere nervousness and fancy. To revert to the case which forms the subject of the present notice. It is clear that it has been in a great degree one of a nervous character, the excessive irritability of the uterus and sensibility of the external parts affording sufficient evidence on that score. But, whether there has been actual uterine disease as well, is difficult, indeed impossible, to be determined. The last miscarriage evidently gave rise to inflammation of the veins, and cannot be supposed to have improved the unfortunate patient's condition.

The preceding case of neuralgia of the uterus is followed by a clever paper on the Unity of Organic Structure, by Dr. Dick, of Clifton. He labours to familiarise his readers with the analogies of formation and structure which exist between different organs of the body. The cranium, for example, has of late been demonstrated as an expanded vertebra, or rather three expanded vertebræ, and the physiologists who have pursued these analogical reveries upon the Continent, are familiarly known by the name of transcendentalists. Their speculations have elicited something that is interesting and curious—much that is fanciful, if not absurd—and little that is likely to be of any use.* The well-informed medical practitioner should not be ignorant of their scientific researches. But we must pass to more practical matter.

### IV. On Hydrometra. By *J. M. Coley*, Esq. of Bridgenorth.

Mr. Coley remarks that dropsy of the uterus is a rare disease, and has been confounded with preternatural secretion from the inner surface of the amnios. His object is to set its pathology in a clearer light, by the narration of two cases. Before we notice them, we may introduce his description of the complaint, which is short, at all events, if not strictly correct.

"When dropsy of the uterus is unconnected with pregnancy, the first obvious symptom, as far as my experience and observation extend, is a frequent discharge of offensive mucus from the vagina, with occasional detachment of organized lymph, in the shape of a membrane, resembling the membrana decidua. On examination, the os uteri is found closed and healthy; and the cervix, in the cases I have met with, greatly and irregularly thickened, indurated and exceedingly tender. The whole course of the vagina is inflamed, and painful on examination. Enlargement of the uterus, from the secretion of fluid within it, follows, which is either absorbed after the cure of the original disease, or the uterine distention is reduced by the spontaneous discharge of its contents.

The original disease appears to be an inflammation in the mucous coat, followed by ulceration. The deposit of morbid secretion in the fibrous coat, when

* It may be well to know that the male and female organs are essentially the same, and that both are merely a repetition of some part of the intestinal canal and its appendages; and it is pleasant to observe how the transcendentalists demonstrate the similarity between the labia and the lips—the clitoris and the tongue—the hymen and the soft palate—the vagina and the gullet—the uterus and the stomach and intestines—the fallopian tubes and the hepatic and pancreatic ducts—the ovaries and the liver and pancreas. The delighted reader rises from the pursuit of such beautiful and simple analogies, with his mind strengthened and refreshed.

it occurs, is probably the consequence of the same specific inflammation. Great pain accompanies the ulcerative process, as in analogous affections, where ulceration of the fibrous structure follows inflammation in its investing membrane." 359.

But, if the original disease be inflammation and ulceration of the mucous membrane, it differs from other mucous membranes in secreting, when so affected, a watery fluid. When we consider, too, how frequent inflammatory affections of the uterine mucous membrane are, and how rare dropsy of the organ is, we must hesitate before we subscribe without reserve to the doctrine on which Mr. Coley leans. This, however, is a theoretical consideration, and we proceed to the cases he brings forward. Before we do so, we must not omit to observe that, as the disease proceeds, the glands of the cervix uteri secrete the gelatinous fluid peculiar to utero-gestation, and the mouth of the organ is sealed. We will take Mr. Coley's second case first, because, being a fatal one, it is satisfactory in a pathological point of view.

*Case* 1.—*Feb.* 12, 1834. A female, æt. 36, mother of two children, the youngest nine years old, had been confined to her bed for four months with a tumor in the region of the uterus, attended with obstinate constipation, hectic fever, and extreme emaciation. On examination, Mr. Coley found a painful, irregular tumor in the hypogastrium, resembling that produced by the uterus in the sixth month of pregnancy, exceedingly tender to the touch, hard and prominent on the left, and comparatively flattened and elastic on the right side of the abdomen. The pain she felt was of a shooting kind, constant and varying in degree of intensity. The os uteri was sound and a little dilated. The cervix was closed and three-fourths of an inch long. The adjoining parts of the distended uterus, within reach of the finger, were of a stony hardness, unequal on the surface, and exquisitely tender, especially on the left side. The vagina was also particularly tender, and during the last four months afforded, at intervals, a dark-coloured, offensive, thick discharge, with portions of a membranous substance. Menstruation had ceased, and the breasts were enlarged and firm. From her own account, it appeared that, a year and a half previously, gradual enlargement of the abdomen commenced, with suppression of the menses; that she then believed herself to be pregnant; and that, at the end of seven or eight months from the commencement of this state, a sudden discharge of offensive fluid, with portions of a membranous substance, proceeded from, and completely reduced the volume of the uterus. Having made preparations for her expected parturition, and been thus deceived, she felt convinced, by her own sensations, that her present symptoms indicated a return of the disease.

Soon after this, Mr. Ingleby, of Birmingham, saw the patient, and thought that the uterus contained a living fœtus, a mistake which he afterwards freely acknowledged. The motion which he had taken for that of the child was the peristaltic action of the intestines. In March, Mr. Coley again saw her. He could discover no fluctuation in the uterus, from the vagina. In the latter end of March, there was a slight hæmorrhage from the vagina, preceded by the detachment of a thick piece of abnormal membrane. About the middle of May, peritonitis occurred. This was followed by purpura; and on the 15th she died.

*Dissection.* This was not performed till the 17th, when there were found :—

" Extreme emaciation. The omentum, in a state of mortification, intimately adhering to the uterus in front; and the abdominal peritoneum, on the left side of the body, was nearly a quarter of an inch thick, and inseparably united to the peritoneal coat of the matrix, which on every part, free from adhesions, was thickened and indurated with lymph, irregular on its free surface, and approaching to a tubercular condition. Every attempt to separate the omentum and abdominal peritoneum from the serous coat of the uterus was prevented by their intimate consolidation. The most firm union, thickening, and induration of these membranes were met with at the part, where the greatest tenderness, hardness, and resemblance to tubercle or scirrhus were observed during life; and that portion of the uterus in front, which had presented through the abdominal parietes the elasticity and figure of the swelling, produced by the serous fluid secreted by the amnios, was free from adhesion, except that with the omentum, which has been already described.

The fibrous portion, or body of the uterus, was so disorganized, that it was not thicker than an ox's bladder, and in some places it was altogether destroyed by an ulcerative process, which had commenced in the mucous membrane. On slight pressure being applied, the peritoneal coat at one spot, being free on both surfaces, gave way, and a thin, dark-coloured, offensive fluid, resembling that which proceeds from an ulcerated intestine, and containing portions of coagulable lymph, to the amount of three pints, escaped. The fibrous coat was quite destroyed at other parts, as well as the spot where the rupture took place; and the uterus, on being divided, collapsed like wet wash-leather, being generally reduced in thickness to the eighth of an inch, and having entirely lost its firmness and elasticity. In short, the principal support and figure of the organ were dependent on its indurated peritoneal coat, except at the inferior part near the cervix.

The whole of the internal or mucous surface of the uterus was found in a state of *ramollissement*, or of that species of ulceration observed in the mucous coat of the intestines in certain fatal diseases of these parts.

The cervix was obliterated with the gelatinous secretion, peculiar to the state of utero-gestation; and the walls of the uterus, adjacent to that part, were enlarged, and consolidated with a tuberculous mass, the principal portion of which was deposited in that part which rested against the rectum, and obstructed its passage. This morbid production consisted of an uniform white structure, and was free from those radiating bands, that gristly feel, and irregular surface, discoverable in scirrhous indurations.

The other abdominal viscera were free from disease, except the colon, which had undergone extreme distention, and was loaded with hardened fæces." 366.

Mr. Coley conjectures that the disease, in this instance, commenced in the mucous membrane of the uterus, and extended from it to the other tissues. When, however, such extensive alterations have occurred, it is difficult to determine how or in what tissue they commenced. Mr. Coley may be right; yet it is not usual to observe such excessive mischief from ulceration of the mucous membrane of the intestines, as he supposes to have followed ulceration of the uterine mucous membrane in the present case.

Mr. Coley refers to two cases lately published, the one by Dr. Ramsbotham, the other by Dr. A. T. Thomson. In the former, the mucous coat of the uterus was entirely destroyed by ulceration, the walls of the organ not being more than one-fourth of their usual thickness, and several black spots, as in his own case, being observable in different parts. In Dr. Thomson's case, as in Dr. Ramsbotham's, the os uteri was obstructed, and, in the latter, almost obliterated. Dr. Thomson also refers to a case, related by Vesalius, in which the uterus was found to contain 180 pints of serous fluid, the os uteri being firmly closed. It is probable that dropsy of the uterus existed, and was cured in a patient, who consulted Dr. Thomson on account of an uterine tumor, which had been gradually increasing during eleven months; and a similar case was observed in the practice of Dr. Merriman.

Mr. Coley details the particulars of a case, which terminated in a favourable manner. This we shall notice.

*Case* 2. On the 31st of March, he was consulted by a female, aged 30, of an exsanguineous complexion, on account of pain in the region of the uterus, accompanied with a sense of prolapsus, and a troublesome discharge of a thin, acrid, mucous fluid from the vagina. She had three times supposed herself to be pregnant while suffering with this disease, and at the end of three months, each time, a flow of an offensive thin fluid took place, followed by an immediate subsidence of the swelling in the hypogastrium. On examination, he found the vagina very tender, and the uterus, adjoining the cervix, so indurated, thickened, and sore, that he suspected the existence of carcinoma. The os uteri, however, was healthy, and he determined to try the effects of mercury. As soon as the mouth became affected, the thickening and induration of the uterus subsided, and at the end of two months, all these symptoms had disappeared. In the following August, there was a slight discharge from the vagina, which was relieved by copaiba, and a lotion of alum and zinc.*

In Dr. Thomson's case, mercurials appeared to be productive of some service.

### V. RECORDS OF OVARIAN TUMORS.

Such is the title of a paper by Dr. Barlow, physician to the Bath Hospital. His object is to add some contributions to the pathology of ovarian tumors, a subject on which our information is neither so precise nor so extensive as we could desire. It is only, as Dr. Barlow observes, by means of multiplied observations, that we can hope to acquire accurate information.

The majority of the cases we are about to notice were those of patients of the Bath Hospital. The records were kept with fidelity and diligence, and Dr. Barlow, having no theories to bolster up, confines himself to simple condensation and analysis. He takes occasion to observe, prior to commencing the narration of the cases, that, in their earlier stages, ovarian tumors probably fail to display the same structural characters and alterations, which we find them exhibit at a late period of the disease; *then* all seems hopeless disorganization. Unfortunately, we are not possessed of a sufficient number of dissections of ovarian tumors in an early stage, to enable us to arrive

* It is common to see copaiba ordered for vaginal discharges. Yet probably it exerts no influence whatever on the mucous membrane of the *vagina*. The practice is drawn from the effects of the medicine on the male urethra—a very different affair.

at any satisfactory conclusions on this point. It is certain that ovarian tumors frequently run a very slow course, and that occasionally they diminish, or even disappear, under the influence of remedies. We have something to hope, besides the gratification of scientific curiosity, from the prosecution of inquiry. We will proceed at once to our author's cases.

Case 1.—*Ovarian Tumor, rapidly fatal.*

Mrs. E. S. æt. 36, had married early and borne six children, the youngest three years old, when, about Midsummer, 1828, she appeared out of health, and the abdomen began to display some fulness. Her indisposition increased, and an examination of the abdomen was made; when a small tumor was distinctly felt, midway between the umbilicus and the symphysis pubis. In a short time, effusion into the abdominal cavity took place—enlargement proceeded rapidly—fluctuation became indistinct, and finally ceased altogether—and in October the patient died. A short period before death an attempt was made to relieve the oppressive distention by tapping, but unsuccessfully. The effusion being then encysted, a single sac only was punctured; but little fluid was drawn off, and the distention was unrelieved. Worn out by suffering and exhaustion, she did not survive the operation many days.

*Dissection.* Nearly the whole abdomen was filled by a large tumor, which pushed up the diaphragm, and displaced the intestines. The peritoneum was inflamed, the viscera being more or less agglutinated by lymph, and the parietes of the abdomen being united to the anterior part of the tumor.

The tumor itself appeared, externally, as one uniform cyst, but when cut into, it was found to be made up of innumerable cysts of different sizes, and in different states; some were not larger than a marble, and contained fluid of a milky whiteness; others contained the same kind of mahogany-coloured fluid that was let out at the operation, with large flakes of lymph mixed with it, approaching the nature of pus; others contained a fluid having the consistence, the appearance, and the odour of pus. Most of those in the centre of the tumor were highly inflamed. Most of the cysts were thick, with large bloodvessels running on their surface, and they were divided and subdivided by cellular bands. The largest cyst would have held eleven or twelve ozs. The left side of the tumor extended into the pelvis; the right side, and the great bulk of the tumor, were elevated above the pelvis. It was the right ovarium, unconnected with any other part, except by the adhesions to the anterior parietes. The right fallopian tube was unaffected, and its fimbriated end was free. The other parts of the uterine system, and the other abdominal and thoracic organs were tolerably sound.

It is obvious that the ovarian tumor, in the preceding case, was the seat of considerable inflammatory action. Inflammation of an ovarian cyst is not an uncommon consequence of tapping it, but occasionally, as in the case before us, it occurs independently of any operation. The medical attendant should be well aware of this, and when he finds that the tumor is tender on pressure, or painful at other times, with a frequent pulse, warm skin, and irritable stomach, he should employ those means which are calculated to reduce, in a gentle manner, inflammatory action.

Case 2.—*Ovarian Tumor, simulating Ascites—Injurious Effects of Mercury.*

Towards the end of 1823, Miss E. S. æt. 22, applied to Dr. Barlow with symptoms of impaired health, and fluctuation in the abdomen, which Dr. B. attributed to ascites. She had been declining in strength for some months, and had been looked on as dyspeptic. Dr. B. prescribed remedies calculated to remove peritoneal effusion, but in a short time peritoneal inflammation supervened. This was relieved, but the enlargement of the abdomen continued, and, indeed, advanced. On the 15th of March she was tapped, and 21½ pints of straw-coloured serum, like that of ascites, were drawn off. The subsequent tappings, the quantities abstracted, and the nature of the fluid, are shewn in this table.

| Date of Tappings. | Quantity. Pints. | Date of Tappings. | Quantity. Pints. |
|---|---|---|---|
| 1825.—15th March, | 21½ | 1826.—4th July, | 17 purulent |
| 13th May, | 10½ | 25th July, | 8 purulent |
| 22d July, | 15 | 22d Aug. | 4 |
| 9th Dec. | 12 | 9th Oct. | 8½ semipurulent. |
| 1826.—24th April, | 27 | 13th Nov. | 11 |
| 10th June, | 21 purulent | 4th Dec. | 13 |

Soon after the last tapping, she died.

*Dissection.* The disease proved to be ovarian, the seat of effusion being one large sac arising from the left ovarium, and extending over the whole abdomen. At the bottom of the sac the ovary was considerably enlarged; and, when cut, exhibited a congeries of sacs of different sizes, filled with all the different secretions which such tumors usually present. The principal sac which covered the several viscera, was but slightly adherent to the parts around, and easily separated, no vascular attachments having taken place.

Dr. Barlow alludes particularly to a circumstance in this case, which is practically not unimportant.

Some time previously to the tapping of June, 1826, the general health was giving way, and disease obviously making more rapid advance. It was very desirable to arrest its progress; and after deliberate consultation, a more active use of mercury was resolved on as worthy of trial. She was confined to bed; took full doses of calomel; and, as the system was not easily affected, inunction was conjoined. The gums became tender, but salivation was not induced. After pursuing this course for a certain time, it was relinquished, not from any ill effects immediately induced, but from the hopelessness of any benefit resulting. Very shortly after, however, the constitution seemed at once to give way; sickness and vomiting occurred, the mouth and fauces were filled with aphthæ, and there was good reason to believe that a similar condition pervaded the lining membrane of the whole alimentary canal. With great difficulty was she recovered from the exhaustion that ensued, bark and wine being largely required. She did recover, however, from the constitutional disturbance; but, on the next ensuing tapping, in June, the contents of the sac, instead of the straw-coloured serum antecedently drawn off, proved to be complete pus, such as any large abscess would yield, and which was evacuated to the extent of 21 pints.

The two following tappings, of the 4th and 25th of July, also yielded purulent matter; after which the purulency abated, and the fluid discharged was noted as semipurulent, but it never recovered its serous character.

The preceding case is an example of the more ordinary form of ovarian dropsy. One cyst acquires a large size, and becomes distended with fluid.

Other small cysts occupy the remainder of the ovary, but do not attain sufficient magnitude to constitute an important portion of the tumor.

Case 3.—*Ovarian Tumor fatal, from Ulceration of the Sac and Effusion of its Contents.*

A servant, æt. 40, became affected, in June, 1831, with pains in the abdomen, quickly followed by swelling of it and of the legs. In July, her size was equal to that of a woman in the eighth month of pregnancy. Her size increased rapidly, with so much tenderness of the abdomen, that blood-letting was necessary. On the 15th she was tapped, and 13 pints of dark puriform fluid, intermixed with flocculi of lymph, were discharged. The improvement after the operation was considerable, but only temporary, for the effusion returned, her health suffered, and, on the 3d of August, she was admitted into the Bath United Hospital.

On the 8th, the abdomen was 38 inches in circumference, with much distress in breathing. She was to be again tapped, but circumstances delayed the operation, and in the night of the 11th, she was seized suddenly with vomiting, giddiness, colliquative sweats, and extreme prostration. At noon next day she died.

On dissection, it was found that an ovarian sac had ulcerated, and that, through the ulcerated aperture, its contents had escaped into the abdomen.

Dr. Barlow observes that this case is calculated to shew us, that delay in tapping an ovarian cyst is not always beneficial.

The two cases which follow in the paper present no particular features to detain us. We, therefore, proceed to some in which the result was not so unfortunate as in those we have hitherto related.

Case 4.—*Ovarian Tumor—beneficial Effects of Antiphlogistic Treatment.*

This case is very long, both in the narration, and in the period it was under observation. The details are spread through a space of three years, and the individual was ten times an out-patient of the hospital, and eight times an in-patient. We must exercise some violence on the particulars, and content ourselves with exhibiting the more prominent. A tableau or two will give a sufficient notion of the more important.

Sophia Wilkins, æt. 21, first became an out-patient in Sept. 1828. She had then febrile complaints, and pain in the lower part of the abdomen, with a very irregular condition of bowels, which were sometimes costive and sometimes relaxed. Antiphlogistic and other appropriate treatment relieved these symptoms, and she quitted the hospital in January.

She had not been long out before she had "epileptic seizures," and those numerous symptoms which, to our mind, prove sufficiently distinctly that she laboured under "hysterical" disorders. She was sometimes an out-patient, sometimes an in-patient, till March, 1830, when she again became the latter. About this time, the hypogastrium had grown so full, and a tumor on the right side was so prominent, that Dr. Barlow applied a caustic issue over it. The effect would appear to have been highly satisfactory, for in June she was discharged "greatly improved." As an out-patient, she was actively bled and cupped. Now a new symptom presented itself—hæmatemesis. Still bleeding, cupping, leeching, frictions with tartar-emetic, were employed. In June, 1831, there was a renewal of pains in the hypogastrium, with dry tongue and loose bowels. Bleeding, cupping, and blistering set her to rights. This was the last time of her being an in-patient. Thrice afterwards she was an out-patient, having, among other symptoms, epistaxis. The usual means had the usual effect, and after another application in April she took her final leave of the hospital.

Dr. Barlow has constructed, with curious diligence, a table of the bleedings, leechings, and blisterings, which this patient underwent. We extract it, for the edification of our readers.

" TABULAR VIEW.

VENÆSECTIO.

| | | | |
|---|---|---|---|
| 1828.—Sept. | 13 | ad oz. | 12 |
| Dec. | 29 | | 10 |
| 1829.—July | 22 | | 12 |
| Aug. | 24 | | 10 |
| 1830.—Jan. | 10 | | 10 |
| Feb. | 20 | | 12 |
| March | 31 | | 10 |
| April | 17 | | 12 |
| Aug. | 18 | | 10 |
| Nov. | 22 | | 10 |
| Dec. | 6 | | 10 |
| 1831.—March | 16 | | 10 |
| — | 26 | | 10 |
| July | 2 | | 10 |
| — | 25 | | 12 |
| Sept. | 17 | | 10 |
| Dec. | 10 | | 10 |
| 1832.—Jan. | 7 | | 10 |
| | | | 190 |

HIRUDINES.

| | | | |
|---|---|---|---|
| 1828.—Sept. | 17 | 8 | temp. |
| Oct. | 18 | 12 | abdom. |
| — | 23 | 12 | — |
| — | 25 | 12 | — |
| Nov. | 14 | 12 | — |
| — | 26 | 6 | temp. |
| 1829.—Jan. | 9 | 8 | — |
| Feb. | 17 | 12 | abdom. |
| Sept. | 1 | 10 | temp. |
| Dec. | 20 | 12 | abdom. |
| — | 24 | 12 | — |
| — | 25 | 12 | — |
| — | 29 | 12 | — |
| 1830.—Jan. | 18 | 8 | circa anum |
| Feb. | 24 | 12 | abdom. |
| April | 1 | 12 | — |
| — | 18 | 12 | — |
| May | 1 | 8 | circa anum |
| — | 3 | 18 | abdom. |
| — | 24 | 12 | — |
| Oct. | 6 | 12 | — |
| Nov. | 5 | 12 | — |
| — | 14 | 12 | — |
| 1831.—Feb. | 13 | 24 | — |
| — | 23 | 18 | — |
| 1831.—March | 1 | 18 | abdomini |
| — | 6 | 18 | — |
| — | 27 | 18 | — |
| July | 22 | 12 | — |
| — | 24 | 8 | circa anum |
| 1832.—Jan. | 4 | 12 | abdom. |
| | | 386 | |

CUCURBITULÆ CRUENTÆ.

| | | | |
|---|---|---|---|
| 1829.—Sept. | 3, | nuchæ, ad oz. | 10 |
| — | 17, | —— | 12 |
| 1830.—May | 12, | —— | 10 |
| Aug. | 7, | —— | 10 |
| — | 21, | —— | 10 |
| Sept. | 1, | abdomini, | 12 |
| — | 8, | —— | 12 |
| — | 18, | —— | 12 |
| Oct. | 7, | —— | 12 |
| — | 11, | —— | 12 |
| — | 14, | supra renes, | 8 |
| — | 29, | abdomini, | 12 |
| Nov. | 1, | nuchæ, | 10 |
| 1831.—March | 9, | —— | 12 |
| — | 25, | abdomini. | 10 |
| May | 7, | —— | 12 |
| June | 29, | —— | 12 |
| July | 6, | —— | 12 |
| — | 16, | —— | 12 |
| — | 21, | —— | 12 |
| Sept. | 14, | —— | 12 |
| | | | 236 |

VESICATORIA.

| | | |
|---|---|---|
| 1828.—Oct. | 11 | lateri |
| Nov. | 1 | abdomini |
| Dec. | 3 | —— |
| — | 19 | —— |
| 1829.—Jan. | 1 | pectori |
| — | 31 | abdomini |
| May | 2 | —— |
| June | 6 | —— |
| 1830.—Jan. | 1 | —— |
| Feb. | 19 | —— |
| Nov. | 15 | lateri |
| 1831.—Mar. | 12 | nuchæ |
| | | 12" |

Thus this young woman lost, in three years, 190 ozs. of blood from the arm—236 ozs. by cupping—had 386 leeches applied to the temples, abdomen, and anus—and smarted or rejoiced under twelve blisters.

Dr. Barlow owns that the merit of the cure belongs almost equally to the patient and physician, for the former decided when the latter was in doubt.

"The intelligence (he says) of this patient often assisted my judgment in regulating her treatment, and determining in what way relief would be best afforded. Oftentimes, when balancing whether to take blood from the arm, or by cup or leech, has she decided me, by stating her own belief of what would benefit most—a persuasion founded on her experience of past treatment, and unbiassed by the apprehension of pain, by which weaker minds would have been influenced. Many times, when local depletion was indicated, have I hesitated to apply cupping-glasses to the hypogastria, lest the pain occasioned should be too severe, when she urged me to disregard such consideration, as she was sure that cupping would relieve her more than any other remedy." 413.

On reverting to the case, we discover no very positive evidence of much ovarian disease; the characters are rather those of a general hysterical or nervous condition, connected, no doubt, in an intimate manner, with disturbance of the uterine system. Obstructed catamenia—epileptic seizures——hæmatemesis—epistaxis—continual vascular disturbances, are symptoms which the practical physician cannot fail to read in the sense we have alluded to. Yet the result of the treatment employed is deserving of consideration, proving, as it does, that dangerous as excessive depletion is in very many of these cases, there are yet some females, in whom it may be practised with great advantage, if it is not absolutely indispensable.

The succeeding cases are brought forward by our author, as remarkable examples of recovery, under circumstances that appeared exceedingly unfavourable.

Case 5. A girl, æt. 18, applied at the hospital on the 11th of October, 1828, on account of abdominal pains and some degree of fever. She had menstruated regularly, but scantily. On the 22d, she was received into the house.

"At this time, the abdomen was very large, and it continued so throughout her illness, at times increased so as greatly to exceed the size of ordinary pregnancy. The fulness, however, though general over the abdomen, had commenced in the iliac region, and ascended; all the evidences derived from the symptoms and the progress of the disease, marking it as decidedly ovarian. On her admission, the torpidity of the bowels claimed the chief attention, the most active purgatives being required. Contingent derangements soon appeared. First affections of the chest with hæmoptysis; afterwards determination of blood to the head, with epileptic seizures. These received their appropriate treatment, which was chiefly depletory, due attention being always given to the primary disease. Leeches and blisters were applied consecutively to the head, chest, and abdomen, repeatedly to the latter, the bowels being steadily purged. Acute symptoms being abated, a trial was given to galvanism, with a view to reduce the special tumours, but with little effect. So inactive were the bowels, that the constant and assiduous use of active purgatives was needed." 423.

We need not particularise the purgatives, which were pretty sharp. In Jan. 1829, she was so much relieved, that she was made an out-patient. Whilst an out-patient, vigorous depletion was still employed, and in May she was dismissed. In June, 1830, she returned with obstinate diarrhœa. This was checked by catechu and copper, but, "the abdominal fulness continuing, and the hypogastric tumors increasing," she was re-admitted as an in-patient in July. The "tumor being chiefly prominent in the right hypochondrium," a caustic issue was made over it. Inflammation now attacked the chest, head, stomach, and liver, and she had an attack of jaundice. She recovered "under active treatment." In August she was discharged, and in September again admitted. She had now epilepsy. Depletion was pursued, and succeeded by the use of iodine. She was again dismissed, again received, and discharged again: but we need not dwell on the repetition of former symptoms and of former treatment. After August, 1830, it would seem that there was "a gradual and progressive decline of the tumor." At Christmas, 1831, she thought proper to marry. She became pregnant, and miscarried between the second and third month. After this she became pregnant again, and in April, 1833, was delivered of a healthy child, after an easy labour.

"I have repeatedly seen her within the last two years, in full activity, and professing to be in perfect health. She is always large in the body; but on examination of the abdomen no trace of tumour was perceptible. From the length of time during which she continued a Hospital patient, she was seen and examined by dozens of medical men, none of whom ever entertained a doubt of the nature or character of her disease. How such a result could have obtained, in a case so apparently inveterate, I am unable to explain." 426.

We must suppose that the medical men could not have been deceived, and that ovarian enlargements really existed. There is one consideration which must tend materially to diminish our surprise at such cases as the preceding. The vascular system of young women is in so uncertain and vacillating a state, that the irregular determinations of blood to certain parts and organs are endless. One day, we see such a patient spitting blood from the lungs—the next, perhaps, voiding it from the bowels—soon afterwards attacked with epileptic fits—then seized with menorrhagia. Yet all this ends in nothing serious, and when menstruation is fairly re-established, these formidable symptoms disappear.

The vascular system plays so very important a part, both in the formation and absorption of morbid depositions, that enlargements of some consequence may take place in so vascular an organ as the ovary, without any irremediable alteration of its structure. The practical surgeon or physician soon ceases to be astonished at any of the vagaries of the female constitution. Her uterine system, the animal in animale, modifies the functions of her other organs, and occasions that host of heterogeneous disorders, that ape all maladies, yet often go as they came, and leave—

Not a wreck behind.

It appears to us that Dr. Barlow is too apt to place to the account of inflammation, these determinations of blood in the female. In his cases, we see a succession of phlegmasiæ, combated with heroic depletion. Without questioning his powers of diagnosis, we think we may be permitted to observe, that pains in this spot or in that, and symptoms which closely simulate those of inflammation of any or of every organ, constantly present themselves in women, independently of actual inflammation. It is true that, in individuals of a full habit of body, a little depletion is sometimes of use, but in many, if not in most, of these cases, it is injurious when carried to any extent.

Case 6. M. A. Scudamore, æt. 21, became an out-patient, July 25th, 1829, for acute pains in the abdomen, which had commenced with diarrhœa. Depletion was adopted, but she was seized with nephritis, and was then necessarily received into the house. Calomel and opium, in addition to the former plan, relieved her, and in September she was discharged well.

The pains soon returned, however, and on the 9th, she was re-admitted an in-patient, the special disease beginning to manifest itself by fulness in both iliac regions. On the 10th it is noted, in the record of symptoms, that these tumors were prominent, and separated by a depressed line extending from the umbilicus to the pubes. Leeches, blisters, calomel and opium, &c. were again resorted to, and again with benefit; for in November she left the hospital.

On the 26th of December she again appeared, with the usual abdominal ailments, accompanied with cough and dyspnœa. The abdomen was more full, the breathing much oppressed, and there was œdema of the lower limbs. All these affections were attended to, but on the 6th of January, 1830, it became necessary again to receive her into the house. On this occasion her sojourn in the Hospital was longer than usual, for she was not again discharged until the 4th of May. The period was marked by continued distress within the abdomen, increased by incidental affections, both of the head and heart. There were several severe epileptic seizures; and inordinate action of the heart was accompanied with acute pain of the left side. For all these depletion was necessary, and freely employed. In April the left hypogastric tumor had become so prominent, that a large caustic issue was made over it. In May she was so much relieved, as to be again made an out-patient.

An out-patient she continued till September, diarrhœa being the prominent symptom. This was not checked till she was again admitted. Then iodine was cautiously tried, but, after a fortnight, pains of the head and side, a hot skin, and inordinate action of the heart necessitated its discontinuance. All these symptoms were mitigated by the usual treatment, and she was dismissed in November, the tumors still remaining.

Why should we hunt the unvarying details? Now the head was affected, now the chest, and now the bowels; depletion being employed for all. In April, 1831, the hypogastric tumors had so advanced, that a caustic issue was made over each. In August they were renewed, from her own conviction of their utility. Paralytic symptoms were now added to the former list, but all amended in the latter part of 1831, and, better still, the tumors were sensibly subsiding. Yet we have alternations of improvement and relapse till September 1833, when she too was a married woman. The remainder of the tale may be told in Dr. Barlow's own words.

"Twice afterwards did she become an out-patient for her ordinary disturbances of health, namely, on the 1st of January, 1834, continuing until the 19th of April; and on the 7th of May, continuing to the 16th of August. By this time she was far advanced in pregnancy, and on the 8th of November, she was safely delivered of three female children, who were baptised by the names of Faith, Hope, and Charity. She suckled the whole for the usual period, and both mother and offspring continued afterwards in perfect health, as I had repeated opportunities of witnessing. She is, I understand, again pregnant, and suffers from some of her old complaints, but is now attended by the accoucheur who put her to bed, and has not latterly come under my observation.

A tabular view of the bleedings, &c., such as was annexed to case VI., is scarcely necessary; yet it may not be uninteresting to state, that in the course of this protracted disease, she was bled 45 times, to the extent of 384 ounces; was cupped 12 times, to 102 ounces; had 408 leeches applied; 16 blisters; and 6 caustic issues, four of these being applied over the left ovarium, and two over the right." 433.

Well might her first and second born be christened with the inspiriting appellations of Faith and Hope. Unless their mother had long been pregnant of both, she could not, we conceive, have been delivered of either. Dr. Barlow may be considered the Father of Charity, for verily his were the works.

### VI. CASE OF FUNGOID TUMOR, SUCCEEDED BY MELANOSIS. By Dr. *Norris*, of Stourbridge.

"On February 6th, 1817," says Dr. Norris," Mr.——, aged 57, of light brown hair and fair complexion, apparently in good health, applied to me in consequence of the inconvenience he felt from a tumour, situated nearly midway between the umbilicus and pubes. He told me there had always been a mole exactly in the same spot, and that, nine months ago, the skin around this congenital mark, assumed a brownish hue, and that from the part thus discoloured a tumour began to arise. On examination, I found the swelling was nearly of half the size of a hen's egg, of a deep brown colour, of a firm and fleshy feel, ulcerated on its surface, which discharged a fetid ichorous fluid. The apex of the tumour was broader than its base. Some few months after the appearance of this tumour, distinct nodules sprung up around it, some with slender necks, others with broader bases. This singular production was at length removed by the knife, and the wound went on favourably, and healed. In less than six weeks the tumour again began to grow from the cicatrized surface, and felt hard and semi-cartilaginous, and very soon minute tubercles, of a livid colour, surrounded the tumour; some of them separated from, and others growing into, each other. Of the latter sort there were, at least, forty in number, forming a mass of disease, extending nearly from the spine of one os ilium to the other, and bearing a resemblance to a large bunch of dark-coloured grapes, some of them flattened on the surface, and of various sizes. The prominent scirrhous-looking tumour occupied the centre; the tubercles already formed progressively increased, while fresh ones arose in the vicinity. The glands in the groin were swollen, and slightly tender to the touch.

This disorganization of parts was effected in two months, and continued to increase after that period. Yet the general health of the patient was not so much impaired as to prevent the regular use of exercise, nor did it interfere with the pursuits of business. Lancinating pains occasionally affected the diseased parts, and an early and continued symptom, was an excruciating pain near the right kidney. The secretion from the kidneys, at times, resembled porter, and deposited a lateritious sediment. At length the constitution began to suffer more severely. Nausea and loss of appetite gradually came on, accompanied by restlessness and excessive depression of spirits. Bluish spots arose in the vicinity of a mole upon the sternum; others appeared, in succession, on the sides of the body, and on the back; and very soon the forehead and scalp were disfigured with the same morbid appearances." 439.

A surgical operation was, of course, out of the question. Dyspnœa and cough now came on, and proved exceedingly distressing. The patient's strength gradually sank—he was tormented with a feeling of heat and fever, although the temperature of the surface did not seem to be increased—and, dropsy supervening, his existence terminated.

*Dissection.* On cutting into the original tumor it displayed throughout a reddish and whitish-brown tint not unlike that of the interior of a nutmeg. The newly-formed tumor and tubera exhibited the same dark-coloured appearance, and on puncturing a considerable number of the tubercles, a thick dark fluid was discharged from them.

On making an incision from the upper extremity of the sternum, and exposing the ribs, a small tubercle was found near the angle of one of them. By a division of this morbid growth, it was evident that the disease was not confined to the periosteum, but had extended to the very substance of the bone. On continuing the incision towards the umbilicus, numerous distinct spots were found dispersed through the cellular substance.

On opening the abdomen, there were tubera of various sizes scattered in profusion in every direction—on the stomach, intestines, mesentery, omentum. The mesenteric glands were much enlarged, and on cutting into them a large quantity of a fluid like tar made its exit. The pancreas, liver, kidneys were affected, especially the liver. The spleen and bladder were the only organs exempt from the disease.

The lungs were, on both sides, thickly mottled, in large and smaller masses, throughout the greater part of their texture. The same mottled appearance was still more vividly displayed on the heart, and the specks were minute, more numerous, and distinct; the heart was almost literally encrusted with them, both externally and internally, and the tubera were from the size of a pin's head, to that of a pea. A few small specimens of the disease were deposited on the inner coat of the descending vena cava, but the arteries did not appear involved in the disease. There was about a quart of fluid in the abdomen; nearly a pint in each bag of the pleura; and, perhaps, two ounces in the pericardium. On dividing the scalp, many of the same diseased appearances were observed, both on the skull-cap, and on the fascia covering the temporal muscles. The dura mater was also studded with them, though much less numerously than the mucous and serous membranes. The ventricles of the brain were nearly filled with fluid. The brain itself was apparently healthy; and with the exception of one speck on the leg, the extremities were free from the disease.

Dr. Norris mentions some circumstances which may lead us to suspect that the disease, in this case, displays some hereditary tendencies. The patient's father had numerous small tumors between the shoulders, which were severally cauterized; and, soon afterwards, he died. The tumor in this patient originated in a mole. His father and brothers had many such, his children are affected with them also, and his youngest son has one in the same place where the disease commenced in himself.*

Dr. Norris observes that there is reason to think that melanosis is of

---

* Since the Father's decease, several of the children have been "suffering with tumors of an alarming aspect."

a kindred genus with fungoid tumor. We think there can be no question of that, melanosis appearing to be little more than a variety of fungoid disease.

When nothing satisfactory can be said on the treatment, perhaps it is better to be silent. As the disease has been attributed to excess of carbon in the blood, it is advised to give as little as possible of those aliments from which carbon is obtained. Salt too is recommended to—do, we hardly know what; for it is not proved that the blood is deficient in saline matters. In truth, such remedies are likely to be mere playthings, and if any thing is to be done it is by iodine, or by some of the more potent medicines.

"Laennec believes the ancients were acquainted with melanosis, and alludes to a case in Haller, where an inky fluid was found in the chest; but is it not probable that this liquiform melanosis is nothing more than a secretion, similar to that we observe in the disease called black vomit? and that true genuine melanosis, like fungus hæmatodes, is altogether a new disease?" 445.

Black vomit is not a disease, but a symptom. We would observe too, that there is little reason to doubt that black vomit is nothing more than effused blood blackened by the free acid of the stomach; it cannot, therefore, be styled a secretion, and Dr. Norris' analogy is defective. It would be useless disputing the antiquity or novelty of melanosis. So far as an opinion goes, we think it is more probable that neither it nor fungus hæmatodes are actually new diseases, but that both have been in former days confounded under the general term of cancer.

This completes our notice of the present volume. There are some clinical reports, which will be found in the department of this Journal set aside for them.

